# Evaluation of effects of small-incision approach treatment on proximal tibia fracture by deep learning algorithm-based magnetic resonance imaging

**DOI:** 10.1515/biol-2022-0624

**Published:** 2023-07-06

**Authors:** Xisheng Li, Huiling Yu, Fang Li, Yaping He, Liming Xu, Jie Xiao

**Affiliations:** Department of Orthopedics, The Second Affiliated Hospital (Jiande Branch), Zhejiang University School of Medicine, Jiande, Hangzhou, 311600 Zhejiang, China

**Keywords:** super-resolution reconstruction algorithm, magnetic resonance imaging, small-incision approach treatment, proximal tibia fracture, clinical effects

## Abstract

In this study, magnetic resonance imaging (MRI) based on a deep learning algorithm was used to evaluate the clinical effect of the small-incision approach in treating proximal tibial fractures. Super-resolution reconstruction (SRR) algorithm was used to reconstruct MRI images for analysis and comparison. The research objects were 40 patients with proximal tibial fractures. According to the random number method, patients were divided into a small-incision approach group (22 cases) and an ordinary approach group (18 cases). The peak signal-to-noise ratio (PSNR) and the structural similarity index (SSIM) of the MRI images before and after the reconstruction of the two groups were analyzed. The operative time, intraoperative blood loss, complete weight-bearing time, complete healing time, knee range of motion, and knee function of the two treatments were compared. The results showed that after SRR, the PSNR and SSIM of MRI images were 35.28 and 0.826 dB, respectively, so the MRI image display effect was better. The operation time in the small-incision approach group was 84.93 min, which was significantly shorter than that in the common approach group, and the intraoperative blood loss was 219.95 mL, which was significantly shorter than that in the common approach group (*P* < 0.05). The complete weight-bearing time and complete healing time in the small-incision approach group were 14.75 and 16.79 weeks, respectively, which were significantly shorter than those in the ordinary approach group (*P* < 0.05). The half-year knee range of motion and 1-year knee range of motion in the small-incision approach group were 118.27° and 128.72°, respectively, which were significantly higher than those in the conventional approach group (*P* < 0.05). After 6 months of treatment, the rate of good treatment was 86.36% in the small-incision approach group and 77.78% in the ordinary approach group. After 1 year of treatment, the rate of excellent and good treatment was 90.91% in the small-incision approach group and 83.33% in the ordinary approach group. The rate of good treatment for half a year and 1 year in the small incision group was significantly higher than that in the common approach group (*P* < 0.05). In conclusion, MRI image based on deep learning algorithm has a high resolution, good display effect, and high application value. The small-incision approach can be applied to the treatment of proximal tibial fractures, which showed good therapeutic effects and a high positive clinical application value.

## 1 Introduction

A proximal tibia fracture is common in youngsters and athletes because of the high intensity and variety of physical activities. The delay in treatment often leads to sequelae, and patients’ normal life and activities are affected [1]. Proximal tibia fracture refers to the fracture at the tibial metaphysis and its proximal side. The structure of the proximal tibia is complex and the anterior is protected by few soft tissues, so injury often occurs. Proximal tibia fracture affects patients’ normal life and is not conducive to travel [2]. Falls and traffic accidents usually result in proximal tibia fractures. The common clinical symptoms among patients with proximal tibia fracture include pain and limited limb activity. In addition, it damages related tissues in children and affects normal physiological functions and physical health [3,4,5]. Proximal tibia fracture not only affects physical exercise and life but also affects patients’ psychological state and normal social activities. Consequently, patients are psychologically stressed and anxious, which is not conducive to the stability of the physical state and the smooth progress of the treatment [6,7]. Hence, early treatment and reduction of proximal tibia fractures are vital for normal psychological function [8].

Proximal tibial fracture has a great impact on patients, and surgical treatment is a common treatment method, with extensive clinical application [9,10,11]. At present, relatively common surgical methods mainly include minimally invasive steel plate treatment and traditional open reduction ordinary plate internal fixation treatment [12,13,14]. Various surgical methods need to be carried out under imaging guidance. With simple operation, less radiation, and fast results, magnetic resonance imaging (MRI) images have a strong diagnostic ability for tissues and positive guiding value, as well as positive clinical value in the treatment and effect evaluation of patients with proximal tibial fractures [15,16]. MRI is widely used in the surgical imaging of proximal tibial fractures. However, due to the rich and complex muscle tissue of the tibia and low resolution, the diagnostic effect needs to be further improved. Deep learning image segmentation of imaging images is to classify every pixel in the image and segment sequential images by constructing and training convolutional neural networks. Image segmentation results only show two-dimensional information and the analysis of details is not clear, so it is necessary to carry out three-dimensional reconstruction of the segmentation results. The 3D reconstruction method can draw the 3D spatial surface of the image, the imaging speed is fast, and it has positive clinical application value. In recent years, artificial intelligence has been widely applied in clinical medicine, providing convenience for clinical diagnosis and treatment [17]. The deep learning algorithm is widely used in the field of medical image processing. It can be applied in image classification and recognition to realize automatic classification and recognition of different diseases and lesions, as well as target detection to realize automatic detection and positioning of specific targets. The deep learning algorithm can also realize the segmentation of different tissues and organs in the image according to the characteristics and structure of the medical image, and carry out 3D reconstruction to realize the three-dimensional presentation and real-time observation of human organs. Deep learning algorithm also has positive application value in the registration and correction of medical images, which can be used to register and correct medical images, thus improving the accuracy and reliability of medical images. Deep learning algorithm plays an important role in the field of medical image processing and provides a lot of useful support for the automatic analysis and diagnosis of medical image. Super-resolution reconstruction (SRR) algorithm based on deep learning has been widely used in medical imaging and achieved great success in the medical field. The deep learning algorithm can transform MRI graphically, improve image resolution and image quality, and has positive application value.

In this research, the clinical effect of small-incision approach treatment on proximal tibia fracture was evaluated through MRI based on a deep learning algorithm. The therapeutic effects on patients in the small-incision approach group and common access group were analyzed, as well as operation time, intra-operative blood loss, and healing of two treatment methods were investigated to provide clinical guidance for the treatment of patients with proximal tibia fracture.

## 2 Materials and methods

### 2.1 Research objects

The subjects of this study were 40 patients with proximal tibial fractures admitted to our hospital from January 2019 to June 2021, who underwent surgical treatment. MRI images based on a deep learning algorithm were used to analyze the clinical efficacy of the small-incision approach and the common-incision approach. Forty patients with proximal tibial fracture were randomly divided into a small-incision approach group (22 cases) and an ordinary approach group (18 cases). General information about the two groups of patients was collected, including gender, average age, and body mass index (BMI). The calculation method of BMI is shown in the following equation:

(1) {\rm{BMI}}=\frac{{\rm{weight}}}{{{\rm{height}}}^{2}},]

where “weight” represents the body weight and “height” represents the height.

In the small-incision approach, a total of 12 cases of male patients and 10 cases of female patients, aged 18–45 years are considered; the average age was 29.75 ± 8.27 years and the average BMI was 22.37 ± 2.48 kg/m^2^. In the ordinary approach group, a total of 10 male patients and 8 female patients, aged from 19 to 43 years were considered; the average age was 29.18 ± 8.22 years and the average BMI was 22.59 ± 2.79 kg/m^2^. All patients were evaluated and followed up 6 months and 1 year after surgery. There were no statistically significant differences in gender, age, and BMI between the small-incision approach group and the general approach group (*P* > 0.05), indicating comparability. This study was approved by the Medical Ethics Committee of the Second Affiliated Hospital.

- Inclusion criteria were as follows: (I) medical records of patients were complete; (II) all patients were diagnosed with proximal tibial fracture; (III) all patients were without MRI contraindications; (IV) patients suffered from no complicated malignant tumor; and (V) patients voluntarily participated in the experimental study and signed the informed consent.
- Exclusion criteria were as follows: (I) medical records of the patients were not complete; (II) patients suffered from complicated vital organ diseases; (III) patients suffered from a genetic disease; (IV) patients suffered from immune system diseases; (V) patients suffered from mental disorders or were unable to communicate normally; and (VI) patients were unwilling to participate in the study.

### 2.2 Methods

#### 2.2.1 Research methods

The small-incision approach method was adopted to treat patients in the small-incision approach group. Patients were assisted to take a supine position and fracture reduction was observed. Then, the articular capsule was cut and the meniscus was suspended. After that, the lateral bone block was opened like a book, and the articular surface was reduced by the tilting method. Next, the articular surface reduction and line of gravity of the lower limb were observed. After a satisfactory reduction, the lateral small-incision approach was fixed with a steel plate. It was appropriately stripped from the attachment site of the muscle. After the successful reduction, traction was maintained. If the knee joint moved well, the incision was thoroughly rinsed and drained with negative pressure. Traditional incision reduction and internal fixation with a plate were performed on patients in the common approach group. MRI images, therapeutic effects, and surgical effects of patients in the two groups were observed.


**Informed consent:** Informed consent has been obtained from all individuals included in this study.
**Ethical approval:** The research related to human use has complied with all the relevant national regulations and institutional policies and is in accordance with the tenets of the Helsinki Declaration, and has been approved by the Medical Ethics Committee of Second Affiliated Hospital.

#### 2.2.2 MRI

All patients underwent routine T1WI, T2WI, and GRE/PDWI scanning followed by 3D-SPGR scanning using a Siemens Skyra 3.0T superconducting MRI scanner. The T2× mapping imaging was performed by 8 echo SE sequence sagittal plane scanning. TR was 1,000 ms, TE was 9.9/19.7/28.6/39.5/49.4/59.2/69.1/79 ms, the layer thickness was 4 mm, the interval was 0.8 mm, the field of vision was 16 cm × 16 cm, the matrix was 288 × 160, NEX = 1, and the scanning time was 244 s.

#### 2.2.3 Image reconstruction

After scanning, the regularized level set algorithm with an improved distance was used to generate the reconstructed image. In this study, a distance regularization level set evolution model (DRLSE) was used. Equation (2) is based on the energy function in the DRLSE model. *ϑ* is set as the image area, and *δ* is a level set function. Among them, *ν* > 0 is the regularization item weight parameters and *A*(*ϑ*) is the regularization item:

(2) \delta ({\vartheta })={\rm{\nu }}{{\rm A}}({\vartheta }){\rm{+}}{{\varepsilon }}_{{\rm{e}}{\rm{xt}}}({\vartheta }).]

The regularization term is given in Equation (3):

(3) A({\vartheta })\left=\underset{\Omega }{{\int }}F\left({\vartheta })|\nabla \varnothing |{\rm{d}}x{\rm{d}}y.]

*A* is the potential function and is expressed as in equation (4):

(4) A\left(a)=\left\{\begin{array}{c}\frac{1}{{\left(2\pi )}^{2}}(1-{\rm{\cos }}(2\pi a)),\hspace{ 1em}a\le 1\\ \frac{1}{2}{(a-1)}^{2},\hspace{ 1em}a\ge 1.\end{array}\right.]

In equation (4), *ϑ*(*x*, *y*) shows |\nabla \varnothing |=1] near the zero level set symbol distance feature but |\nabla \varnothing |=0] away from the location of the zero level set.

*ε*_ext_(*ϑ*) refers to the external energy, as expressed in the following equation:

(5) {{\varepsilon }}_{{\rm{ext}}}({\vartheta })=\gamma {W}_{z}({\vartheta })+{\mu }{B}_{z}({\vartheta }).]

{W}_{z}({\vartheta })] and {B}_{z}({\vartheta })] are given in equations (6) and (7), respectively.

(6) {W}_{z}({\vartheta })=\beta \delta ({\vartheta })|\nabla \varnothing |{\rm{d}}x{\rm{d}}y,]

(7) {B}_{z}({\vartheta })=\beta H(-{\vartheta }){\rm{d}}x{\rm{d}}y.]

*β* is an edge index function, based on the edge information symbol balloon force, and the regional information symbol balloon force has been integrated into the DRLSE model. *β* is defined as follows:

(8) \beta =\frac{1}{1+{|\nabla {X}_{\sigma }\times I|}^{2}},]

where {X}_{\sigma }] is the standard deviation *σ* on the number of Gaussian kernel functions. The final energy function is defined as follows:

(9) {\varepsilon }({\vartheta })={\rm{\nu }}{{\rm A}}({\vartheta })+\gamma {W}_{z}({\vartheta })+{\mu }{B}_{z}({\vartheta }).]

### 2.3 Observation indicators

The peak signal-to-noise ratio (PSNR) and structural similarity index (SSIM) of the SRR MRI images were analyzed, which can be calculated by equations (10) and (11), respectively:

(10) {\rm{PSNR}}=10{\rm{lo}}{{\rm{g}}}_{10}\left\{\frac{{{(2}^{m}\left-1)}^{2}}{{\rm{MSE}}}\right\},]

(11) {\rm{SSIM}}(x\left,y)=\frac{(2{{\nu }}_{x}{{\nu }}_{y}+{K}_{1})(2{\sigma }_{{xy}}+{K}_{2})}{({{\rm{\nu }}}_{x}^{2}+{{\rm{\nu }}}_{y}^{2}+{K}_{1})({\sigma }_{x}^{2}+{\sigma }_{y}^{2}+{K}_{2})}.]

In the above equations, MSE represents the measure of error between the reconstructed image and the original image; *x* and *y* represent the image to be tested; {{\nu }}_{x}] and {{\nu }}_{y}] represent the average gray level of the two images, respectively; and *K*_1_ and *K*_2_ are constants.

The operation time and intra-operative blood loss among patients with proximal tibia fractures treated with two therapies were compared.

The full load time and complete healing time among patients with proximal tibia fractures treated with two therapies were compared.

The knee range of motion among patients treated with two therapies half a year and 1 year after surgery was compared.

The effects of two therapies on knee joint functions among patients with proximal tibia fracture half a year and 1 year after surgery were compared.

According to Kolment therapeutic effect assessment criteria, the therapeutic effect was excellent if the knee joint was fully extended with a range of motion greater than 120° without pain. The effect was good if the knee joint was fully extended with the range of motion between 90 and 120° with no or occasional mild pain. The effect was intermediate if the range of motion was greater than 60° with frequent mild pain. The effect was poor if the range of motion was less than 60° with frequent pain or constant pain.

The excellent rate is calculated as shown in equation (12). Excellent, good, and total represent the number of patients with excellent effects, the number of patients with good effects, and the total number of patients, respectively:

(12) {\rm{Excellent\; rate}}=\frac{{\rm{Excellent}}+{\rm{Good}}}{{\rm{Total}}}.]

### 2.4 Statistical methods

Excel 2016 was adopted to record and summarize data, and SPSS 20.0 was employed for data statistics and analysis. Mean ± standard deviation (*X* ± *S*) denoted measurement data, which were analyzed using the *t-*test. Percentage (%) was the representation of count data. *χ* ^2^ test was used, and *P* < 0.05 was considered to have a statistical difference.

## 3 Results

### 3.1 Analysis of MRI images of patients with a proximal tibia fracture

**Figure 1:**
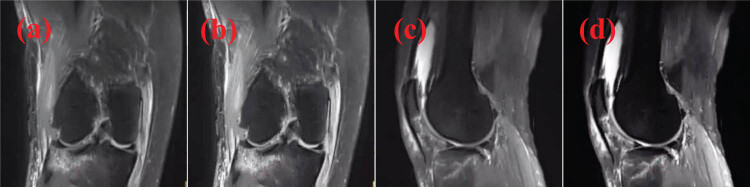
Analysis of MRI images of a male patient aged 37 years with proximal tibia fracture (a and c: original images; b and d: SRR MRI images).

The four MRI images of a patient with proximal tibia fracture are shown in Figure 1 (a and a are original images and b and d are SRR MRI images; b and d have higher resolution and better effect).

### 3.2 PSNR and SSIM of MRI images before and after SRR

**Figure 2:**
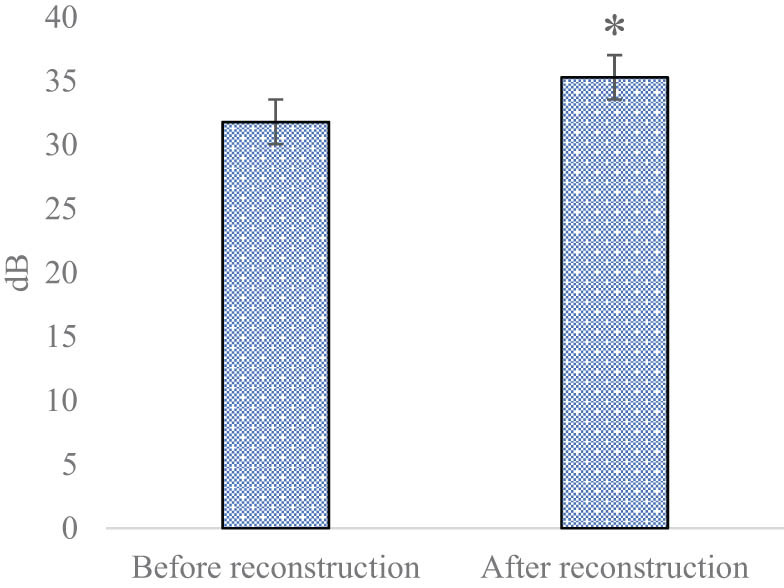
PSNR of MRI images before and after SRR (*P < 0.05 compared with the value before reconstruction).

**Figure 3:**
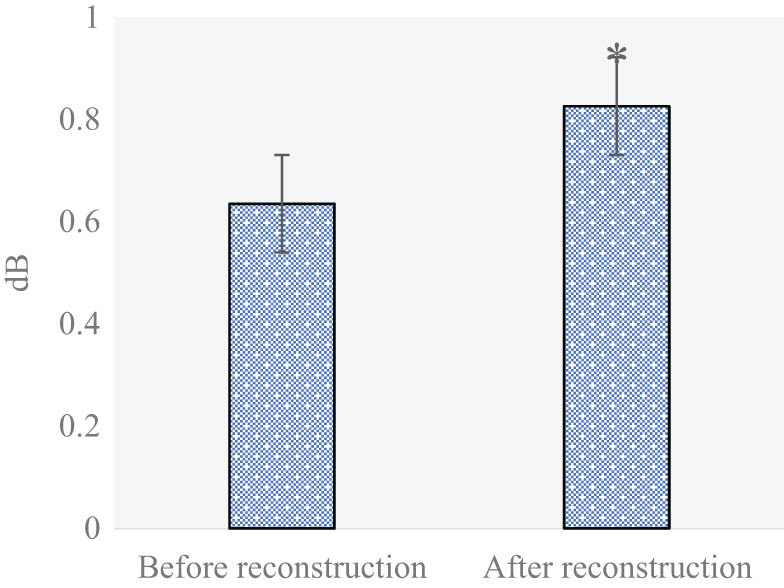
SSIM of MRI images before and after SRR (*P < 0.05 compared with the value before reconstruction).

Figures 2 and 3 show the PSNR and SSIM of MRI images before and after SRR. Before SRR, the PSNR and SSIM of the obtained images were 31.79 and 0.635 dB, respectively; while those after SRR were 35.28 and 0.826 dB, respectively.

### 3.3 Comparison and analysis of operation time and intra-operative blood loss among patients with proximal tibia fracture treated with two therapies

**Figure 4:**
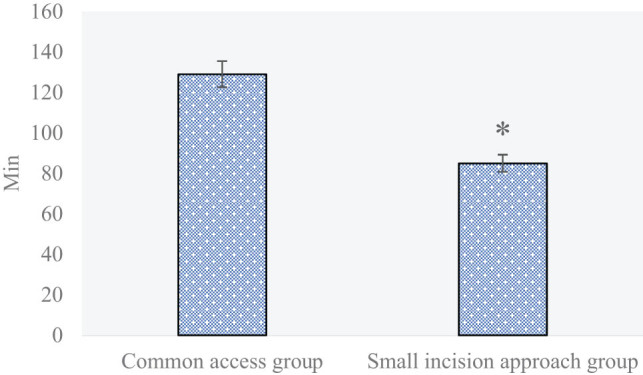
Comparison and analysis of the operation time among patients with proximal tibia fracture in the two groups (*P < 0.05 shows the difference between the two groups).

**Figure 5:**
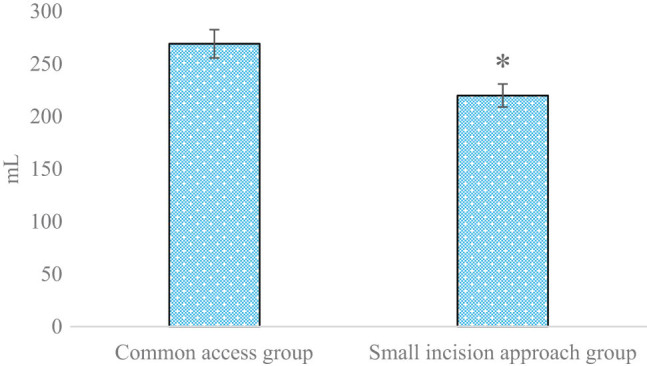
Comparison and analysis of the intra-operative blood loss among patients with proximal tibia fracture in the two groups (*P < 0.05 shows the difference between the two groups).

The operation time among patients with proximal tibia fracture treated with two therapies is compared in Figure 4. The operation times among patients in the small-incision approach group and the common approach group were 84.93 and 128.96 min, respectively (*P* < 0.05). The intra-operative blood loss among patients with proximal tibia fracture treated with two therapies is compared and analyzed in Figure 5. The intra-operative blood loss values among patients in the small-incision approach group and the common approach group were 219.95 and 269.23 mL, respectively (*P* < 0.05).

### 3.4 Comparison and analysis of the full load time and complete healing time among patients with proximal tibia fracture in the two groups

**Figure 6:**
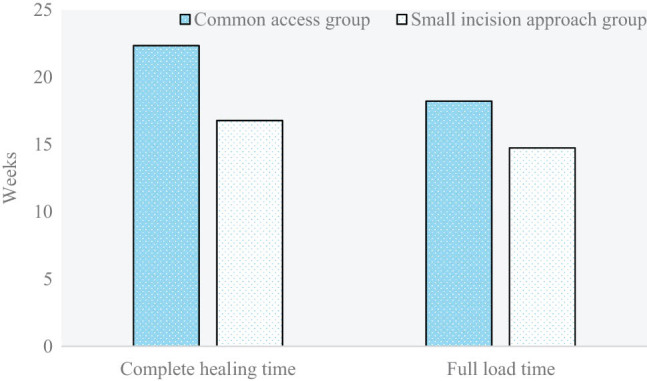
Comparison and analysis of the full load time and complete healing time among patients with proximal tibia fracture in the two groups (*P < 0.05 shows the difference between the two groups).

The full load time and complete healing time among patients with proximal tibia fracture in the two groups are compared and analyzed in Figure 6. Full load times among patients in the small-incision approach group and the common approach group were 14.75 and 18.22 weeks, respectively (*P* < 0.05). Complete healing times among patients in the small-incision approach group and the common approach group were 16.79 and 22.35 weeks, respectively (*P* < 0.05).

### 3.5 Comparison and analysis of the knee joint range of motion among patients with proximal tibia fracture in the two groups half a year and 1 year after surgery

**Figure 7:**
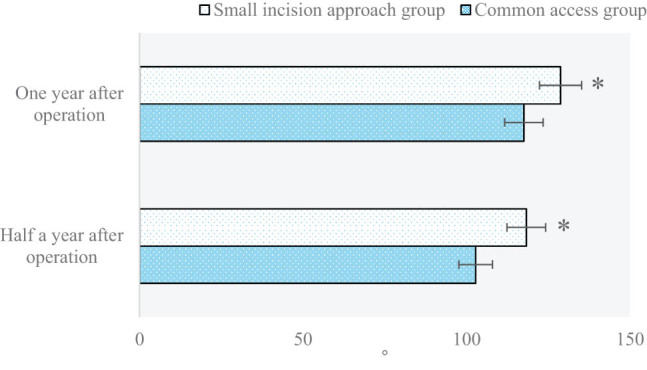
Comparison and analysis of the knee joint range of motion among patients with proximal tibia fracture in the two groups half a year and 1 year after surgery (*P < 0.05 shows the difference between the two groups).

The knee joint range of motion among patients with proximal tibia fracture in the two groups half a year and 1 year after surgery are compared and analyzed in Figure 7. The knee joint range of motion values among patients in the small-incision approach group and the common approach group half a year and 1 year after surgery were 118.27° vs 102.78° and 128.72° vs 117.52°, respectively (*P* < 0.05).

### 3.6 Comparison and analysis of the therapeutic effects on knee joint functions among patients with proximal tibia fracture in the two groups half a year and 1 year after surgery

**Figure 8:**
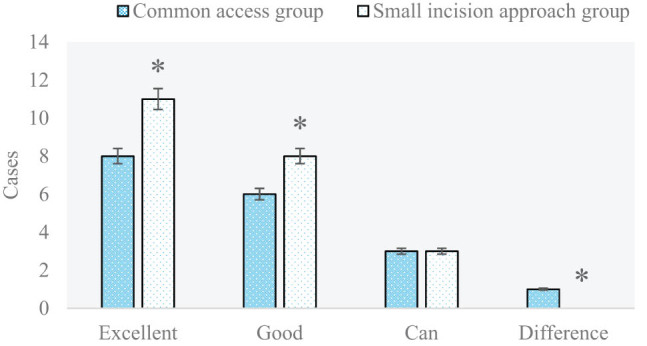
Comparison and analysis of the number of patients with different therapeutic effects on knee joint functions in the two groups half a year after surgery (*P < 0.05 shows the difference between the two groups).

**Figure 9:**
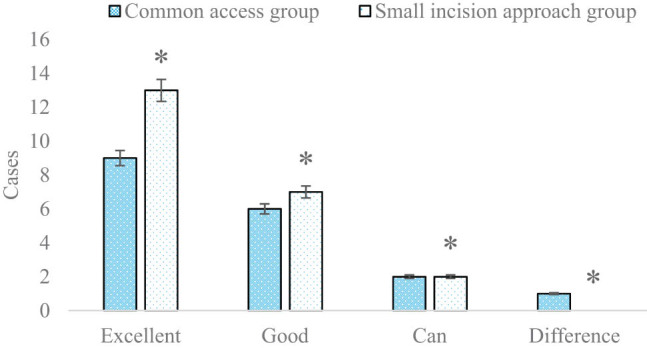
Comparison and analysis of the number of patients with different therapeutic effects on knee joint functions in the two groups 1 year after surgery (*P < 0.05 shows the difference between the two groups).

**Figure 10:**
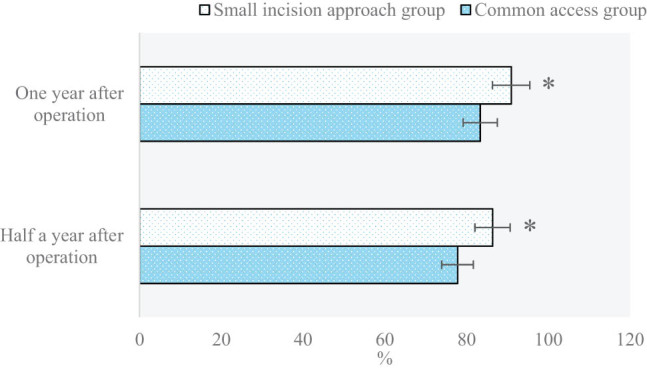
Comparison and analysis of excellent rates of therapeutic effects on knee joint functions among patients in the two groups half a year and 1 year after surgery (*P < 0.05 shows the difference between the two groups).

The number of proximal tibia fracture patients with different therapeutic effects on knee joint functions in two groups half a year and 1 year after surgery is compared and analyzed in Figures 8 and 9, respectively. In addition, excellent rates of different therapeutic effects on knee joint functions in two groups half a year and 1 year after surgery are compared and analyzed in Figure 10. In the small-incision approach group and the common approach group, the number of patients with excellent, good, intermediate, and poor therapeutic effects was 11 vs 9, 8 vs 6, 3 vs 2, and 0 and 1, respectively, half a year after surgery. In the above two groups, the number of patients with excellent, good, intermediate, and poor therapeutic effects was 13 vs 9, 7 vs 6, 2 vs 2, and 0 vs 1, respectively, 1 year after surgery. Excellent rates in the small-incision approach group and the common approach group were 86.36 and 77.78%, respectively, half a year after surgery; and those in the two groups reached 90.91 and 83.33%, respectively1 year after surgery (*P* < 0.05).

## 4 Discussion

A proximal tibia fracture is common, and the disease will aggravate if it is not controlled and improved in time. Consequently, patients’ motor function is seriously affected [18]. The main treatment and improvement methods include surgical therapy and conservative therapy. Surgical therapy is featured with a highly effective rate, rapid symptom relief, and good prognosis [19]. As a minimally invasive treatment method, the small-incision approach therapy is featured with high feasibility, few complications, strong patient autonomy, high comfort, saving time and costs, and few damages to patients [20,21]. As a method for diagnosing proximal tibia fracture and assessing therapeutic effect, MRI imaging possessed remarkable application values. It could be adopted to evaluate the dislocation and limb functions among patients, which provided some references for the assessment of proximal tibia fracture [22,23].

MRI analysis of proximal tibial fracture based on a deep learning model is conducive to improving the therapeutic effect, promoting the recovery of patients, and providing manipulative support for disease control. The results of this study showed that after SRR, the PSNR and SSIM of the obtained images were 35.28 and 0.826 dB, respectively, showing a good application effect. The small-incision approach treatment causes less damage to the joints and muscles of patients, has short operation time and less intraoperative blood loss, which can effectively improve the condition of patients with proximal tibial fracture, reduce the incidence of complications, protect the limb functions of patients, and improve the satisfaction of patients [24,25]. Locking plates are commonly used for the internal fixation of closed tibial fractures. The use of locking plates as external fixators remains controversial, especially for closed fractures. Studies have shown that the small-incision approach is of positive value in the treatment of closed tibial segmental fractures, and the average healing time of proximal fractures is shorter than that of distal fractures, showing better clinical effects [26]. This study analyzed the operative time, intraoperative blood loss, and healing of the two treatments. The results showed that the operation time of patients in the small-incision approach group was significantly shorter than that in the common approach group (*P* < 0.05), and the intraoperative blood loss in the small-incision approach group was significantly less than that in the common approach group (*P* < 0.05). The complete weight-bearing time of the small-incision approach group was significantly shorter than that of the common approach group (*P* < 0.05). The complete healing time in the small-incision group was significantly shorter than that in the common approach group (*P* < 0.05). The half-year knee motion and 1-year knee motion in the small-incision approach group were significantly higher than those in the common approach group (*P* < 0.05). Studies have shown that the use of the small-incision approach to treat proximal tibial fractures has good results, safety and reliability, the least complications, good prognosis, and convenient operation [27]. After half a year of treatment, the rate of good treatment was 86.36% in the small-incision approach group and 77.78% in the ordinary approach group. After 1 year of treatment, the rate of good treatment was 90.91% in the small-incision approach group and 83.33% in the ordinary approach group. The rate of good treatment in the small-incision approach group was significantly higher than that in the ordinary approach group (*P* < 0.05). It can be concluded that the small-incision approach can effectively treat the proximal tibial fracture and relieve the condition of vertebral patients, which has a positive clinical application value.

## 5 Conclusion

The application of a deep learning algorithm to evaluate the clinical effect of the small-incision approach in the treatment of proximal tibial fracture by MRI imaging can provide a reference for the treatment of patients with proximal tibial fracture and have high clinical application value. Therefore, it exhibited a positive significance and can be used in clinics. The shortcoming of this study was that it did not compare and analyze the effect of MRI images of other parts after SRR, which needed further research and verification. The significance of this study was to provide guidance for imaging analysis of patients with proximal tibial fractures in clinical practice and improve the diagnostic effect. In the future, MRI images of other parts can be reconstructed with super-resolution, which proved the clinical value of the deep learning algorithm applied to MRI images.
